# Analysis of Thrombin-Activated Platelet-Derived Exosome (T-aPDE) Potential for Dental Pulp Regeneration: *In-Vitro* Study

**DOI:** 10.1055/s-0042-1744370

**Published:** 2022-06-21

**Authors:** Dini Asrianti Bagio, Indah Julianto, Anggraini Margono, Endang Suprastiwi

**Affiliations:** 1 Department of Conservative Dentistry, Faculty of Dentistry, Universitas Indonesia, Jakarta, Indonesia; 2 Department of Dermato Venereology, Faculty of Medicine, Universitas Sebelas Maret, Solo Surakarta, Indonesia

**Keywords:** platelet, exosome, thrombin, dental pulp, stem cells

## Abstract

**Objective** This study analyzed the potential of various concentrations of the thrombin-activated platelet-derived exosome (T-aPDE) to regenerate the dental pulp by performing an *in-vitro* analysis of the cell viability, migration activity, and vascular endothelial growth factor A (VEGF-A) expression of human dental pulp stem cells (hDPSCs).

**Material and Methods** The hDPSCs were collected from nine third molar teeth of nine healthy donors and were isolated and cultured using the explant method. They were harvested between the third and fourth passages and starved, after which they were seeded in the following treatments: Dulbecco's Modified Eagle Medium and 10% platelet-rich plasma-thrombin as the control groups, and 0.5, 1, and 5% T-aPDE as the experimental groups. All groups had three biological triplicates (Triplo) and two number of experiments. The T-aPDE was analyzed using transmission electron microscopy testing, particle size analyzer, and CD63 +  and CD81 +  specific immune phenotyping flow cytometry tests for plasma exosomes. The cell viability was evaluated using the colorimetric assay of activity cellular enzymes (MTT assay); the migration activity, using scratch assay; and the VEGF-A expression, using enzyme-linked immunosorbent assay.

**Results** The highest viability absorbance value of hDPSCs after 24, 48, 72 hours of observation was in the 5% T-aPDE group ( *p* <0.05). Whereas, the closest distance result of migratory activation hDPSCs was also in the same group ( *p* <0.05). However the highest VEGF-A expression of hDSPCs was noted in the same group at 72 hours observation ( *p* <0.05).

**Statistical Analysis** The data were analyzed using one-way analysis of variance and the Kruskal–Wallis test. The statistical power was set at *p* <0.05

**Conclusion** The 5% T-aPDE had a higher potential to induce dental pulp regeneration than the other groups.

## Introduction

A modified paradigm of free-based cell concepts has been developed over the past few years. 1 2 The American Association of Endodontists approved this therapy concept and created protocols that include cell homing, migration, and attracting factors for endogenous human dental pulp stem cells (hDPSCs) and apical papilla stem cells. 3 4 Nevertheless, studies are still trying to find a suitable conditioned medium for hDPSCs, which is one of the crucial factors that will back up this endogenous stem cell recruitment and growth and provide a favorable microenvironment or niche biology for dental bioengineering. 4 5

Many studies have proven the effectiveness of platelet-based secretomes as supplemented conditioned media for hDPSCs. Studies have verified the potential effectivity of 10% platelet-rich plasma (PRP), 10% human platelet lysate (hPL), concentrated growth factor, 5 to 25% advanced platelet-rich fibrin (A-PRF), 5 to 25% platelet-rich fibrin lysate (PRF-L), and 10% platelet-rich fibrin exudates (PRF-E) for migration, proliferation, differentiation, and angiogenesis of hDPSCs *in vitro* . 6 7 8 9 10 11 12 13 Nonetheless, there have been reports of the potential bias of studies on platelet-based conditioned media, which may explain the variation in the results and may alter the performance of such media in the clinical setting. 14

The exposure of human dental pulp to hypoxic conditions caused by increased reactive oxygen species (ROS) has resulted in increased levels of the transcription factor hypoxia-inducible factor 1 and the vascular endothelial growth factor (VEGF). 15 16 A very high and unregulated ROS level may lead to mitochondrial dysfunction followed by DNA damage, which can cause decreased VEGF expression of hDPSCs within 72 hours. 15 16 17 VEGF (also known as *VEGF-A* , the VEGF family) plays an important role in regulating the pulp vascular permeability and migration of pulp endothelial cells. 16 17 A previous study demonstrated that platelet-based conditioned media can release the platelet-derived growth factor (PDGF) that synergistically upregulates VEGF. 18 Thus, further proof of a change in the VEGF expression of hDPSCs cultured in platelet-based media is important.

In recent years, research has been growing on exosomes as platelet-based conditioned media. They have been reported as a stable form of platelet-based conditioned media and as the effector of secretomes as well as new potential carriers of bioactive proteins, mRNAs, and miRNAs that play crucial roles in cell-to-cell communication. 19 20 21 22 Exosomes are nanosized (≤150 nm) vesicles that have exosomal cargo consisting of biomolecules located inside the vesicles and secreted by almost all metabolically active cells and plasma. Exosomes have specific transmembrane proteins, such as CD9 + , CD63 + , and CD81 + . 23

It has been demonstrated that platelet-based exosomes can be taken up by bone mesenchymal stem cells (BMSCs) after 20 hours and accumulated in the perinuclear region, which had been believed to be related to the role of exosomes in recipient cells. 24 Another study has proven that platelet-rich plasma exosomes (PRP-E) can act as carriers of growth factors. Thus, they have been presented as a novel treatment for osteoarthritis. 25 Later, it was also proven that PRP-E can induce proliferation and migration of endothelial cells that trigger the re-epithelialization of chronic wounds *in vitro* . 26

In dentistry, dental pulp stromal cell (DPSC) exosomes have been studied recently. Their benefits have been shown in many severe dental diseases, 27 and they have also been reported to have the ability to increase the proliferation and the odontogenic differentiation of DPSCs. 28 29 30 Despite this finding, the use of exosomes derived from platelet-based media is believed to be more potential than the use of exosomes derived from stem cells because platelet-based media are more economical and easier to source to obtain large numbers of exosomes. Therefore, this study was the first that analyzed the importance of determining the suitable concentration of thrombin-activated platelet-derived exosome (T-aPDE) for the dental pulp that has the potential to induce dental pulp angiogenesis, an important stage in dental pulp regeneration that is supported by the ability of T-aPDE to fix the mitochondria cell function, through *in-vitro* analysis of the cell viability, migration activity, and VEGF-A expression of hDPSCs.

## Material and Methods

This study was approved in writing by the ethical committee of the Faculty of Dentistry, University of Indonesia (No. 82/ *ethical approval* /FKGUI/IX/2019; Protocol No. 070940819). The informed consent of all the adults who participated in this study was obtained prior to the study.

### Dental Pulp Cell Culture

The hDPSCs that were used in this study were collected from nine third-molar teeth of nine healthy donors who fulfilled the inclusion criteria of this study (age range of donor 18–25 years old, no systematic disease, healthy, and no smoking/alcohol consumption habit). The hDPSCs were isolated and cultured using the explant method, based on a previous study. 31 The hDPSCs were harvested between the third and fourth passages (P3 and 4) and, then, starved for 24 hours in Dulbecco's Modified Eagle Medium (DMEM; Thermo Fisher Scientific Inc., MA, United States) supplemented with 1% fetal bovine serum (FBS). Then, the hDPSCs were seeded in different treatments in the following groups: (the negative control group) (1) hDPSCs + DMEM; (the positive control groups) (2) hDPSCs + DMEM supplemented with 10% FBS; (3) hDPSCs + 10% platelet-rich plasma-thrombin (PRP-T); (the experimental groups) (4) hDPSCs + 0.5% T-aPDE; (5) hDPSCs + 1% T-aPDE; and (6) hDPSCs + 5% T-aPDE. All the groups had three biological triplicates (Triplo). The hDPSCs were analyzed via flow cytometry mesenchymal stem cells (MSCs) analysis using FACSverse (BD Biosciences) with an MSC-positive cocktail (CD90 + , CD105 + , and CD73 + ) and a negative cocktail (Lin ^Neg^ ) for hDPSCs.

### Thrombin-Activated Platelet (from PRP) Preparation

**Fig. 1:**
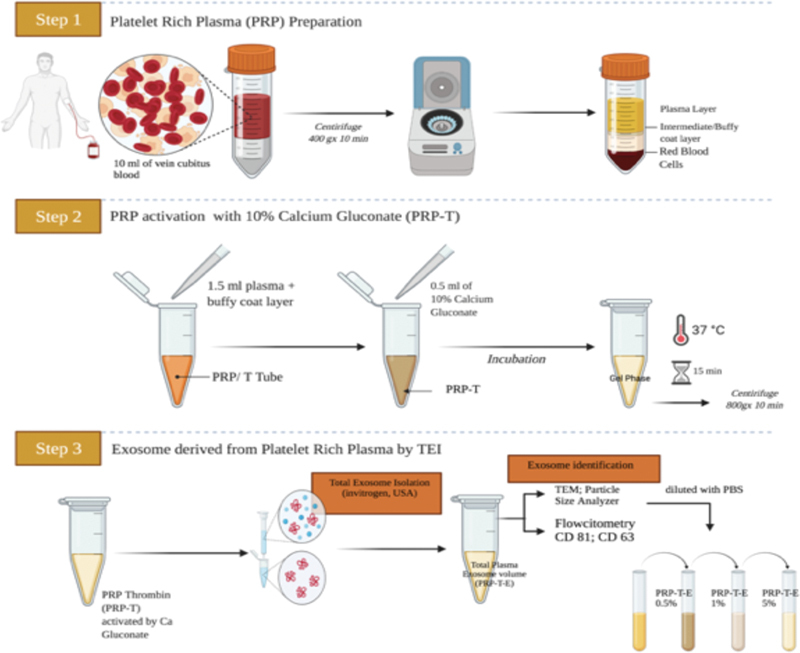
Workflow of the isolation of the thrombin-activated platelet-derived exosome (T-aPDE). The exosome derived from the activated platelet-rich plasma-thrombin (PRP-T) was stated as T-aPDE isolated by the total exosome isolation method, and then, it was divided into three concentrations.

The first step in the T-aPDE preparation was the PRP preparation. The PRP preparation method was used by Franco et al (2012) ( Fig. 1 ). 32 Blood was collected from three donors who had a normal red blood cell (RBC) count (hemoglobin = 13–15 g/dL; hematocrit = 38–46%; and erythrocytes = 3.8–5.2 × 10 ^6^ /μL; laboratory results attached) and who matched the following inclusion criteria for this study: age, 12 to 18 years, healthy without any systemic disease; not taking aspirin; no smoking habit; and no alcohol consumption. Only 10% of the total plasma volume of the thrombin-activated platelet (from the PRP) (10% PRP-T) was used in this study.

### T-aPDE Isolation

The T-aPDE (from the PRP) was transferred to a new tube, and 0.5 mL of phosphate-buffered saline (PBS; calcium-free, then magnesium-free, and phenol red-free; Gibco, Thermo Fisher Scientific Inc.) was added to it. Next, 3 µL of a total exosome isolation (TEI) (protein precipitation) reagent was added to the tube (Invitrogen, Carlsbad, CA, United States) ( Fig. 1 , step 3). 33 The reagent and the PRP were mixed until they were homogeneous, using a vortex or a pipette tip. Then, the mixture was incubated at room temperature for 10 minutes, after which it was immediately centrifuged at 10,000 g for 5 minutes at room temperature. The supernatant was aspirated and disposed of with a pipette (the visible pellets at the bottom were exosomes) and, then, centrifuged again at 10,000 g for 30 seconds to remove debris. The supernatant was again aspirated and removed carefully with a pipette supernatant. Then, 1x PBS was added to the platelet pellet and mixed using a vortex for resuspension. The resuspension was stored at 4°C to −20°C before use. The total plasma protein volume of the exosomes that were used in this study was divided into the following concentrations: 5, 10, and 50 µg/mL (0.5, 1, and 5%) based on previous study ( Fig. 1 ). 26

### T-aPDE Identification: Size and Morphology

**Fig. 2:**
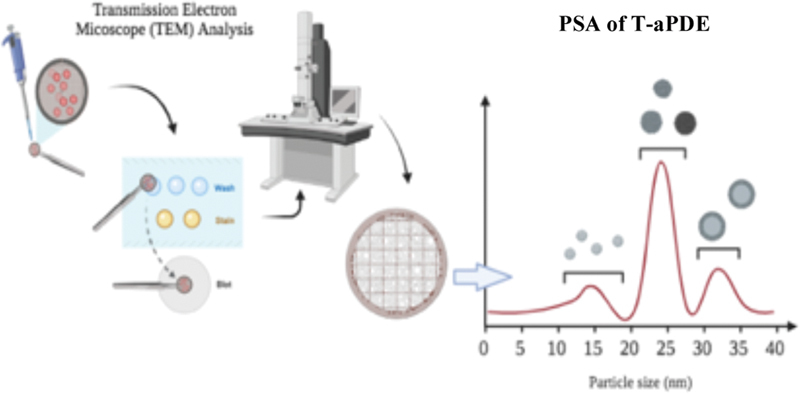
Determination of the size and morphology of the T-aPDE.

The sizes and morphologies of the T-aPDE were analyzed using a particle size analyzer (PSA) and transmission electron microscopy testing (TEM; Hitachi TEM System, HF-3300). 34 For the TEM analysis, a few steps were conducted. First, the T-aPDE pellets and the supernatant were fixed with 1 mL of 2.5% glutaraldehyde (pH 7.0) for 1 hour at 4°C. Second, the pH was adjusted to 7.4 using 0.1-M hydrogen chloride and dissolved with 200 mL of distilled water. Third, the 2.5% glutaraldehyde was removed, and the T-aPDE pellets and the supernatant were washed with 1 mL of PBS (Gibco, PBS solution, Thermo Fisher, United States) solution at room temperature. The pellets and the supernatant were incubated for 10 minutes with 90% acetone with propylene oxide, cleaned, and incubated again for 30 minutes. Staining was done using 2% uranyl acetate for 20 minutes and lead nitrate for 10 minutes. The T-aPDE pellets and supernatant were fixed with epoxy resin after the dehydration and staining stages and, then, cut into a thin film layer (40–150 nm). The resulting film specimens were stored in a grid box for TEM at 80 kV ( Fig. 2 ).

### T-aPDE Immunophenotyping

The physical characteristics of the T-aPDE were analyzed with standard identification methods using an exosome-specific surface marker test (CD63+ and CD81+; ab267479 Exosome Isolation and Analysis Kit—Flow Cytometry, Plasma, Abcam, Shanghai, China), 34 following the manufacturer's instructions.

### Viability Assay of the hDPSCs

The viability assay test of the hPDSCs in this study used 96 wells (5 × 10 ^3^ cells/well) of 3-(4,5-dimethiazole-2-yl)2,5-diphenyltetrazolium bromide (MTT) assay read with a microplate reader at an absorbance of 595 nm. The viability of the hDPSCs in 0.5, 1, and 5% T-aPDE; in the positive control (10% PRP-T); and in the negative control (DMEM) was evaluated after 24, 48, and 72 hours in terms of the absorbance value of their MTT assay. The conversion of the absorbance value of the MTT assay resulted in the percentage of the hDPSC viability rate. The formula for the conversion absorbance value, also known as the optical density (OD), from the MTT assay to the percentage of the viability rate of the cells (%) is as follows: 35



### Migration Activity of the hDPSCs

The migration activity of the hDPSCs was analyzed based on the wound closure area of the hDPSCs and the closure speed rate/wound with the hDPSCs using the scratch assay. 36 The differences in the migration activity of the hDPSCs were analyzed by comparing the migration activity of the hDPSCs that were cultured on 0.5, 1, and 5% T-aPDE and on 10% PRP-T with the migration activity of the control group (DMEM).

Then, the hDPSCs were incubated in 5% CO _2_ atmosphere at 37°C for 24 hours, after which their development was observed with a microscope. The resulting migration was photographed three times sequentially per well using a digital photographic graph (Zeiss Observer Z1 microscope, UK). The size of the wound/scratch area and the speed rate were calculated with Image J software and statistically analyzed.

### VEGF-A Expression of the hDPSCs

The quantitative evaluations of the VEGF-A expression of the hDPSCs in 0.5, 1, and 5% T-aPDE were compared with those of the control group (10% PRP-T) using a human VEGF-A enzyme-linked immunosorbent assay (ELISA) kit (Cat. E-EL-H0111, Elabscience, Wuhan, Hubei). The expression was measured after 24, 48, and 72 hours, following the manufacturer's protocol, on an ELISA microplate reader under a wavelength of 405 nm, a detection range of 31.25 to 2,000 pg/mL, and a sensitivity value of 18.75 pg/mL.

### Statistical Analysis

The hDPSC viability absorbance values from the MTT assay and the migration activities of the hDPSCs were compared with one-way analysis of variance (ANOVA), migration activity of the wound closure area and the speed of the wound closure (speed rate) of the hDPSCs were tested for reliability using the intraclass correlation coefficient (ICC) and compared with one-way ANOVA, and then the VEGF-A expressions of the different groups were compared using the Kruskal–Wallis test followed by the *post hoc* Mann–Whitney test. All the tests were conducted at a significance level of 95% ( *p* < 0.05). All the data were analyzed using IBM SPSS Statistics Software, version 22.0 (IBM Corp., Armonk, NY, United States).

## Results

### Characterization of the hDPSCs

**Fig. 3:**
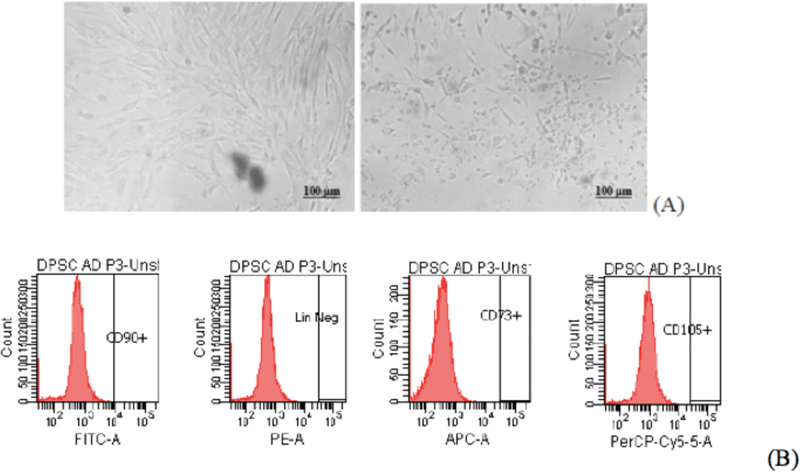
Results of the **(A)** harvesting of the human dental pulp stem cells (hDPSCs) between the third and fourth passages (P3 *–* P4) ( *left* ) followed by their 24-hour starvation in Dulbecco's Modified Eagle Medium (DMEM) supplemented with 1% fetal bovine serum (FBS; right) and of the **(B)** flow cytometry mesenchymal stem cells marker expression of the hDPSCs used in this study, showing a positive cocktail of CD90+ (97.9%), a negative cocktail of Lin ^Neg^ (0.5%), CD105+ (97.7%), and CD73+ (98.6%).

The qualitative and MSC marker expressions of the hDPSCs in this study are presented in Fig. 3 . The hDPSCs were homogeneous and spindle-shaped and had looser colonies ( Fig. 3A , left).

### Characterization of T-aPDE: Size, Morphology, and Immunophenotyping

**Fig. 4:**
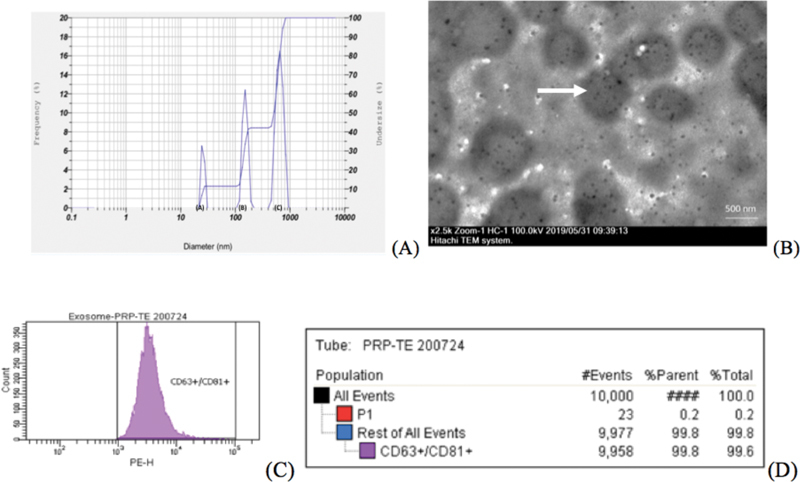
Sizes and morphologies of T-aPDE by transmission electron microscopy: particle size analyzer results. **(A)** A double lipid layer of exosome vesicles was seen at the outer part: vesicles ( *white arrow* ) and exosomes ( *white arrow* ). **(B)** Results of the physical characterization analysis of PRP-E protein using the specific immunophenotyping flow cytometry test for plasma (ab267479 Exosome Isolation and Analysis Kit—Flow Cytometry, Plasma, Abcam) for the T-aPDE of CD63+ and CD81+ ( **C** – **D** ).

The PSA results showed a mean range and distribution of 44 to 127 nm in the exosome pellet body of the T-aPDE, and the TEM results showed morphology of T-aPDE ( Fig. 4A ).

### Viability Assay of the hDPSCs after Culture in PRP-T and 0.5, 1, and 5% T-aPDE

**Fig. 5:**
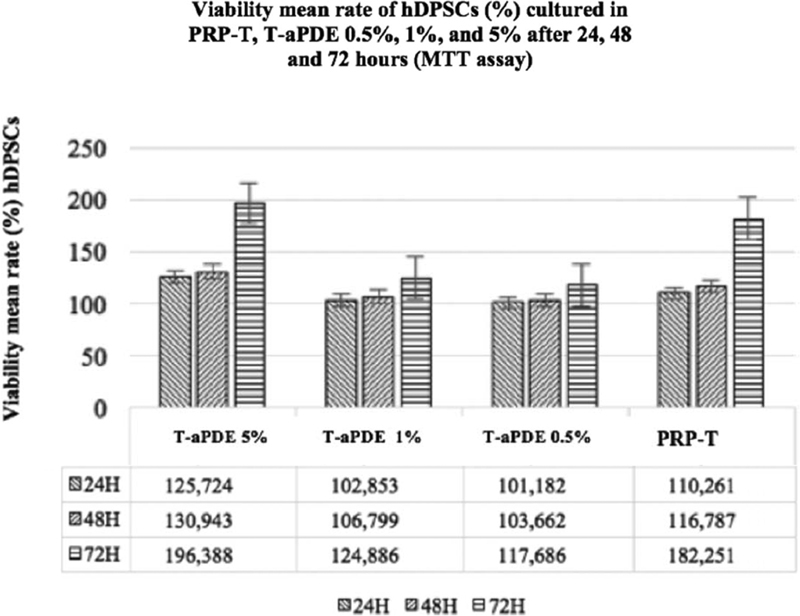
Viability mean rate of the viable hDPSCs (%) cultured in PRP-T and T-aPDE (0.5, 1, and 5%) after 24, 48, and 72 hours of observation (and after conversion of the absorbances values of the 3-(4,5-dimethiazole-2-yl)2,5-diphenyltetrazolium bromide assay to the percent of viable cells).

The viability assay of the hDPSCs cultured in 0.5, 1, and 5% T-aPDE; 10% PRP-T, and control (DMEM) was performed using an MTT assay kit (Sigma-Aldrich, Cat. No. 11 465 007 00, Roche) at a wavelength of 595 nm. It showed that after 24 to 72 hours of observation, the hDPSCs with the highest absorbance value were noted in the 5% T-aPDE group and there were significant differences between all the groups ( *p* < 0.05; one-way ANOVA). The OD and the percentage (converted from the OD) of the viable hDPSCs cultured in different culture media from the MTT assay are presented in Fig. 5 . The figure shows that the OD of the 5% T-aPDE group was statistically more significant than those of the other groups after 24 and 48 hours of observation, but there was no significant difference between the OD of the 5% T-aPDE group and that of the 10% PRP-T group after 72 hours of observation.

### Migration Activity of the hDPSCs after Culture in PRP-T and 0.5, 1, and 5% T-aPDE

The intra-rater observation data on the differences in the migration activities of the hDPSCs in terms of the wound closure area and the speed of the wound closure (speed rate/wound width) were tested for reliability using the ICC. The results showed that the reliability ( *r* ) = 0.976 a, which is higher than the *r* value. Thus, it can be concluded that the observation data on the migration activities of the hDPSCs are reliable.

**Fig. 6:**
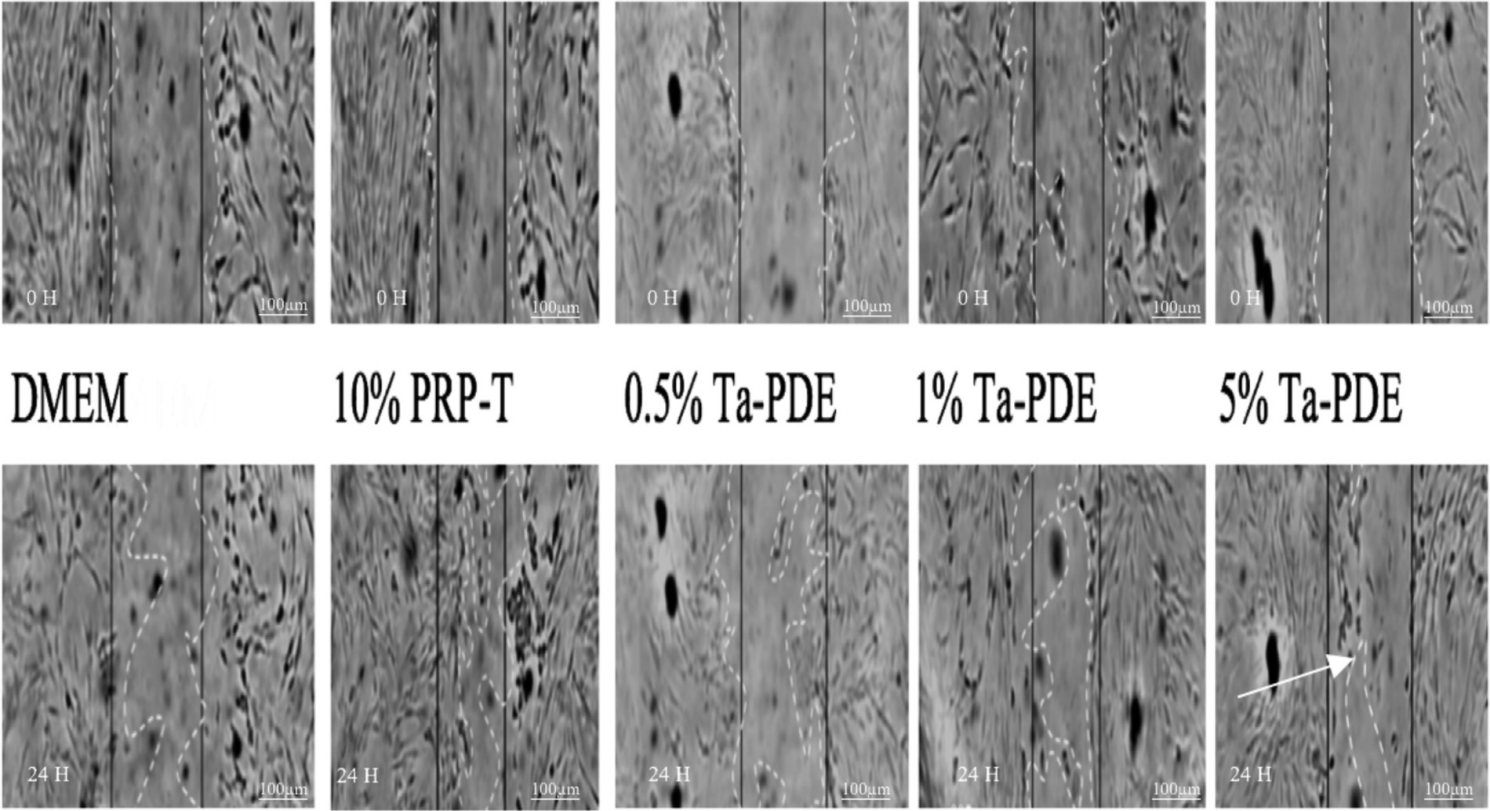
Qualitative results of the migration activity of the hDPSCs showing that the 5% T-aPDE group had the highest wound closure area (97.52%) and the fastest speed rate (3.069 μm/h) compared with the other groups ( *white arrow* ) after 24 hours of observation.

The migration activities of the hDPSCs cultured in PRP-T and 0.5, 1, and 5% T-aPDE are presented in Table 1 . There are significant differences in the means for the wound closure (%) area and the speed rate (micrometer per hour) of the hDPSCs in 0.5, 1, and 5% T-aPDE and in 10% PRP-T compared with the control group after 24 hours ( *p* < 0.05; one-way ANOVA). The quantitative results of the migration activities of the hDPSCs are presented in Fig. 6 .

**Table 1:** Analysis of differences in the migration activities of hDPSCs evaluated by means of the wound closure area and the speed rate at various concentrations of T-aPDE (0.5, 1, and 5%) and of the control group after 24 hours

Conditioned media	Wound closure area (%)	Speed rate (μm/h)
	24 h	
	Mean ± SD	Mean ± SD
DMEM	41.887 ± 11.168	0.087 ± 0.104
10% PRP-T	83.095 ± 19.891	1.844 ± 0.150
0.5% T-aPDE	48.685 ± 4.402	0.937 ± 0.401
1% T-aPDE	57.700 ± 2.445	1.147 ± 0.125
5% T-aPDE	97.520 ± 0.425	3.069 ± 0.845
*p* -Value	0.000 a	0.000 a

Abbreviations: ANOVA, analysis of variance; DMEM, Dulbecco's Modified Eagle Medium; hDPSC, human dental pulp stem cells; PRP-T; platelet-rich plasma-thrombin; SD, standard deviation; T-aPDE, thrombin-activated platelet-derived exosome.

a One-way ANOVA test, *p* <0.05.

### VEGF-A Expressions (Pictogram per Milliliter) of the hDPSCs in Various Concentrations of T-aPDE after 24, 48, and 72 hours of Observation

**Fig. 7:**
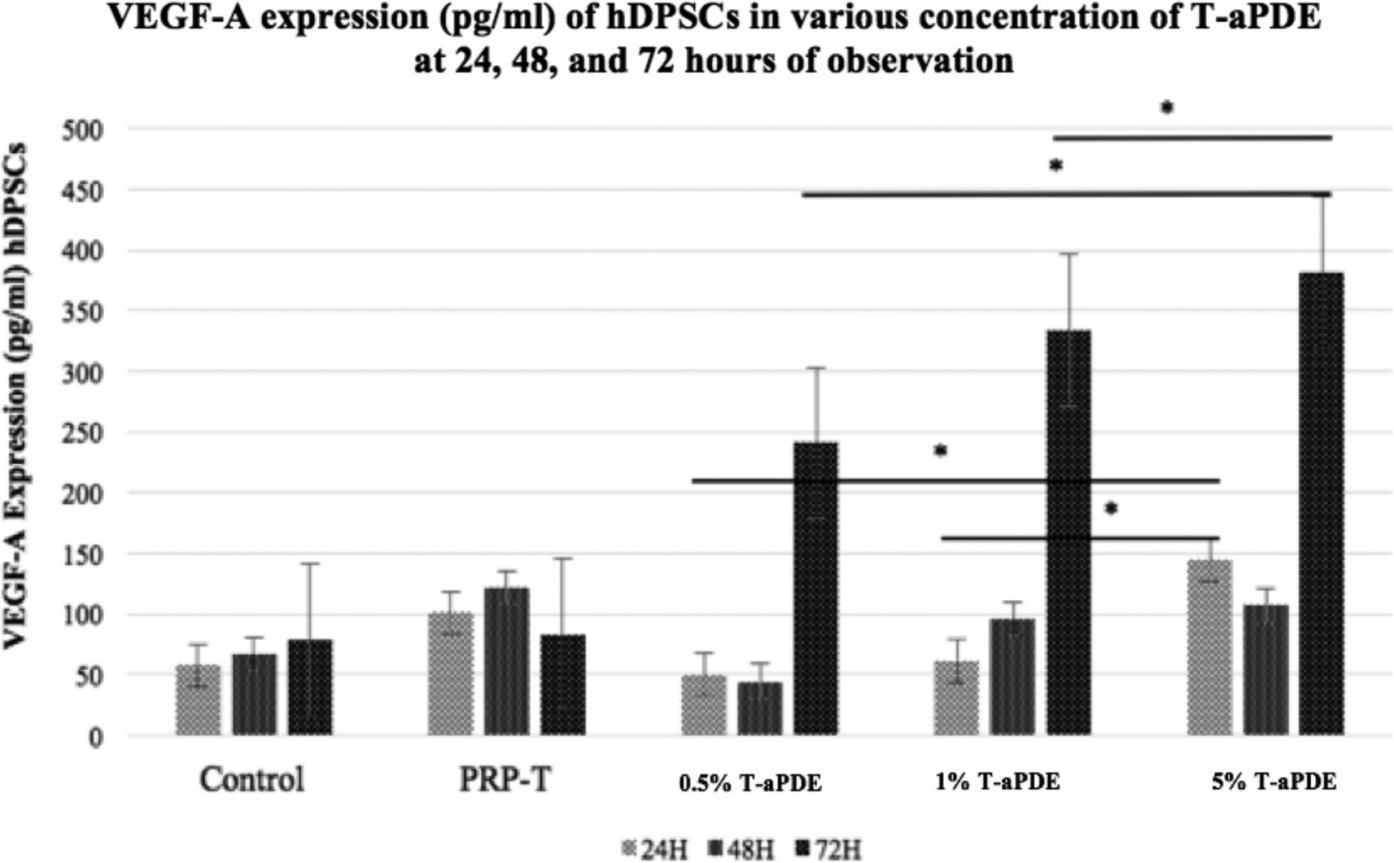
Quantitative results of the *post hoc* vascular endothelial growth factor A expressions of the hDPSCs after their culture in T-aPDE groups (0.5, 1, and 5%) and comparison with 10% PRP-T at 24, 48, and 72 hours of observation. The *post hoc* analysis of the T-aPDE groups showed statistically significant differences between 5% T-aPDE and the control group and other concentrations of T-aPDE after 24 and 72 hours of observation ( *p* < 0.05) ( *post hoc* Mann–Whitney test). * *p* < 0.05; ns = not significant.

The VEGF-A expressions of the hDPSCs in 0.5, 1, and 5% T-aPDE after 24, 48, and 72 hours of observation differed significantly from those of the positive control group (10% PRP-T) ( *p* < 0.05; Kruskal–Wallis test, *p* < 0.05). The *post hoc* analysis results are presented in Fig. 7 (horizontal line: significant comparison of the groups by dose and time).

## Discussion

The characteristics of the hDPSCs used in this study are shown in Fig. 3 . They are correlated with the results of a previous study that revealed that hDPSCs obtained using the explant and enzymatic methods were comparable in terms of the flowcytometry marker expression of their MSCs. 31 37

The PRP that used in this study were collected from three donors, related to the numbers of experiment on this study and triplicate group samples procedures. Although the PRPs were collected from three donors, the differences between the donors, from their laboratory examinations, were within the normal range, besides which they had the same blood type and their ages were close to each other. These conditions for obtaining valid research results had been determined from a systematic review of a PRP study that discussed how to reduce potential bias in research using PRP so that the research results would become valid. 14

In this study, the isolation of T-aPDE corresponded with a previous study that also used the TEI reagent method ( Fig. 1 ). 33 34 38 According to the standard regulations of the International Society for Extracellular Vesicles, at least two different technologies must be used to identify exosomes. 34 In this study, morphology was performed using TEM and particle size analysis (nanometer) was performed using a PSA ( Fig. 4A, B ), followed by the specific surface expression of immunophenotyping using flow cytometry (CD63+ and CD81+). The morphology of the exosomes using TEM showed a double lipid layer with exosomes as black dots ( Fig. 4B ). This result is similar to the results of other exosome studies. 39 40 From the PSA analysis of the T-aPDE pellets, the sizes of the exosomes were found to have a mean range and distribution of 44 to 147 nm ( Fig. 4A ). This result is in accordance with that of a previous study that revealed that the size of exosomes from ultracentrifugation may be more homogeneous than the sizes obtained from other methods. 39

Flow cytometry showed that 99.9% of the exosome population (P1) expressed CD63 +/CD81+ in the T-aPDE used in this study ( Fig. 4C, D ). However, this result is not in line with that of another study that exosomes isolated from TEI may show less surface expression specific for exosome markers. 38 Therefore, it can be concluded from the T-aPDE samples in this study that most of their protein particles could be defined as exosomes derived from PRP-T, unlike in previous studies, where fewer protein particles of exosomes were generated from the TEI method. 38 39

It was previously demonstrated that 10% PRP, 10% hPL, 5 to 25% A-PRF, 5 to 25% PRF-L, and 10% PRF-E have a potential ability to support hDPSC proliferation and migration, as well as odontogenic differentiation and angiogenesis. 6 7 8 9 10 11 12 13 14 However, clinical application of these platelet-based secretomes is often constrained by their heterogeneous results and bias potential. 14 It is thought that PRP in exosome form is more stable and can be stored for longer periods than regular PRP. 21 22 In this study, the T-aPDEs were stored at 4°C for 7 days prior to the start of the experiment, but the results were still in line within each steps (data not shown). This finding was also correlated with that of a previous study that proved that saliva exosomes could be stored at 4°C for up to 25 days 20 and at −80°C for more than a month. 22

Cell viability test has been determined as a parameter of healthy cells in populations. 35 In this study, the resulting absorbance values of the viable cells in the 10% PRP-T and 0.5, 1, and 5% T-aPDE groups compared with those of the control group revealed that the 5% T-aPDE group could produce hDPSCs with a superior mean viability rate compared with the other conditioned media culture groups ( Fig. 5 ). This result is consistent with the findings of a previous study that PRP-Es have better fibroblast cell activity than activated PRP-As. 26 Other studies revealed better proliferation of rabbit chondrocyte cells in a 50-µg/mL dose of PRP-Es and proliferation of BMSCs in a 50-µg/mL dose of platelet lysate exosomes. 25 These findings are also correlated with the results of a previous study that demonstrated that exosomes can be taken up by MSCs and found a perinuclear region of MSCs after 20 hours that was believed to have been related to the intracell re-program mechanism of miRNA exosomes in their recipient cells. 24 In this study, the 5% T-aPDE group showed superior viability after 24 hours, which remained stable and developed until after 72 hours ( Fig. 5 ). This result indicates that the 5% T-aPDE culture media group was able to maintain more viable hDPSCs and potentially induced cell proliferation.

The MTT assay was conducted by measuring the colorimetric substrate of the mitochondria enzymes of the cell. 35 The mitochondria function improved after the hDPSCs were seeded in the 5% T-aPDE medium, compared with the control groups ( Fig. 5 ). Although after 72 hours, the absorbance value of the PRP-T group after the MTT assay was comparable to that of the 5% T-aPDE group, the 5% T-aPDE group still had a higher absorbance value up to 72 hours. This phenomenon can be related to the potential of the cargo-containing miRNA from T-aPDE to reduce the ROS in the hypoxic pulp. (In this study, the hDPSCs were first starved 24 hours before their culture in the experimental media.) 15 40 This ROS reduction mechanism was created by the specific capability of T-aPDE to work on cell nuclei intracellularly and then to epigenetically reprogram the cell to repair its DNA before fixing the re-function of the mitochondria cell. 40 The results of the cell viability test in this study were in line with the results on the migration activity of the hDPSCs ( Table 1 ), which showed that the 5% Ta-PDE group had the best migration activity of all the groups ( Fig. 6 ), although further research is needed to prove this finding.

The angiogenesis process of hDPSCs is one of the key factors of dental pulp tissue regeneration, and VEGF-A is one of the main growth factors (GFs) related to this process. 16 In a previous study, human dental cells were able to express VEGF 24 to 72 hours after a wound occurred. 17 This study demonstrated that the VEGF-A expressions of hDPSCs escalated more significantly 24 to 72 hours after they were cultured in 5% T-aPDE than after they were cultured in the control medium (DMEM), 10% PRP-T, and other T-aPDE media (0.5 and 1%) (see Fig. 7 ). This finding also indicates that 5% T-aPDE can release PDGF, which synergistically upregulates VEGF, resulting in higher VEGF expression after 72 hours ( Fig. 7 ). 18

It has been reported that several factors of research on platelet-based conditioned media (PRP and PRF) have the potential to cause bias, including in relation to the blood donors for the PRP preparation (i.e., their age, gender, RBC/platelet counts, collection technique, and time), the cells used, the observation time, and the activation agent. 14 41 Therefore, the use of PRP as the basic platelet material of T-aPDE can cause study limitations. However, even though the potential for bias in this study was minimized, it is strongly recommended that the results of this study be applied based on the materials and methods used in this study. 14

## Conclusion

In conclusion, this study strongly showed that 5% Ta-PDE has the potential to induce dental pulp angiogenesis, an important stage in dental pulp regeneration, due to the ability of Ta-PDE to fix the mitochondria cell function, as revealed by the results of the *in-vitro* analysis of the cell viability and the migration activity of the hDPSCs. This novel finding is expected to be the starting point for determining the suitable T-aPDE concentration for dental pulp. Further studies need to be conducted on the complete mechanism of dental pulp regeneration that can be induced by T-aPDE.
